# Advancements in extracellular vesicle targeted therapies for rheumatoid arthritis: insights into cellular origins, current perspectives, and emerging challenges

**DOI:** 10.1186/s13287-024-03887-x

**Published:** 2024-09-04

**Authors:** Maryam Talebi Jouybari, Fatemeh Mojtahedi, Mahnaz Babaahmadi, Maryam Faeed, Mohammadreza Baghaban Eslaminejad, Leila Taghiyar

**Affiliations:** 1 Department of Stem Cells and Developmental Biology, Cell Science Research Center, Royan Institute for Stem Cell Biology and Technology, Banihashem Square, Banihashem St., Resalat Highway, PO Box: 16635–148, Tehran, 1665659911 Iran; 2 Department of Developmental Biology, University of Science and Culture, Tehran, Iran; 3 Advanced Therapy Medicinal Product Technology Development Center (ATMP-TDC), Royan Institute for Stem Cell Biology and Technology, ACECR, Tehran, Iran; 4 Department of Immunology, Shahid Sadoughi University of Medical Science, Yazd, Iran; 5 School of Biology, College of Science, University of Tehran, Tehran, Iran

**Keywords:** Rheumatoid arthritis (RA), Extracellular vesicles (EVs), Exosomes, EV-based therapies, Drug delivery

## Abstract

Rheumatoid arthritis (RA) remains a challenging chronic autoimmune disorder characterized by persistent joint inflammation and damage. While modern regenerative strategies, encompassing cell/stem cell-based therapies, gene therapy, and tissue engineering, have advanced tissue repair efforts, a definitive cure for RA remains elusive. Consequently, there is growing interest in developing targeted therapies that directly address the underlying mechanisms driving RA pathogenesis, such as extracellular vesicles (EVs). These small membrane-bound particles can modulate immune responses within the inflammatory microenvironment of damaged cartilage. To launch the clinical potential of EVs, they can be isolated from various cell types through several techniques. EVs can carry various bioactive molecules and anti-inflammatory or pro-regenerative drugs, deliver them directly to the affected joints, and affect the behavior of injured cells, making them a compelling choice for targeted therapy and drug delivery in RA patients. However, there are still several challenges and limitations associated with EV-based therapy, including the absence of standardized protocols for EV isolation, characterization, and delivery. This review provides a comprehensive overview of the cellular sources of EVs in RA and delves into their therapeutic potential and the hurdles they must overcome.

**Supplementary Information:**

The online version contains supplementary material available at 10.1186/s13287-024-03887-x.

## Introduction

**Fig. 1:**
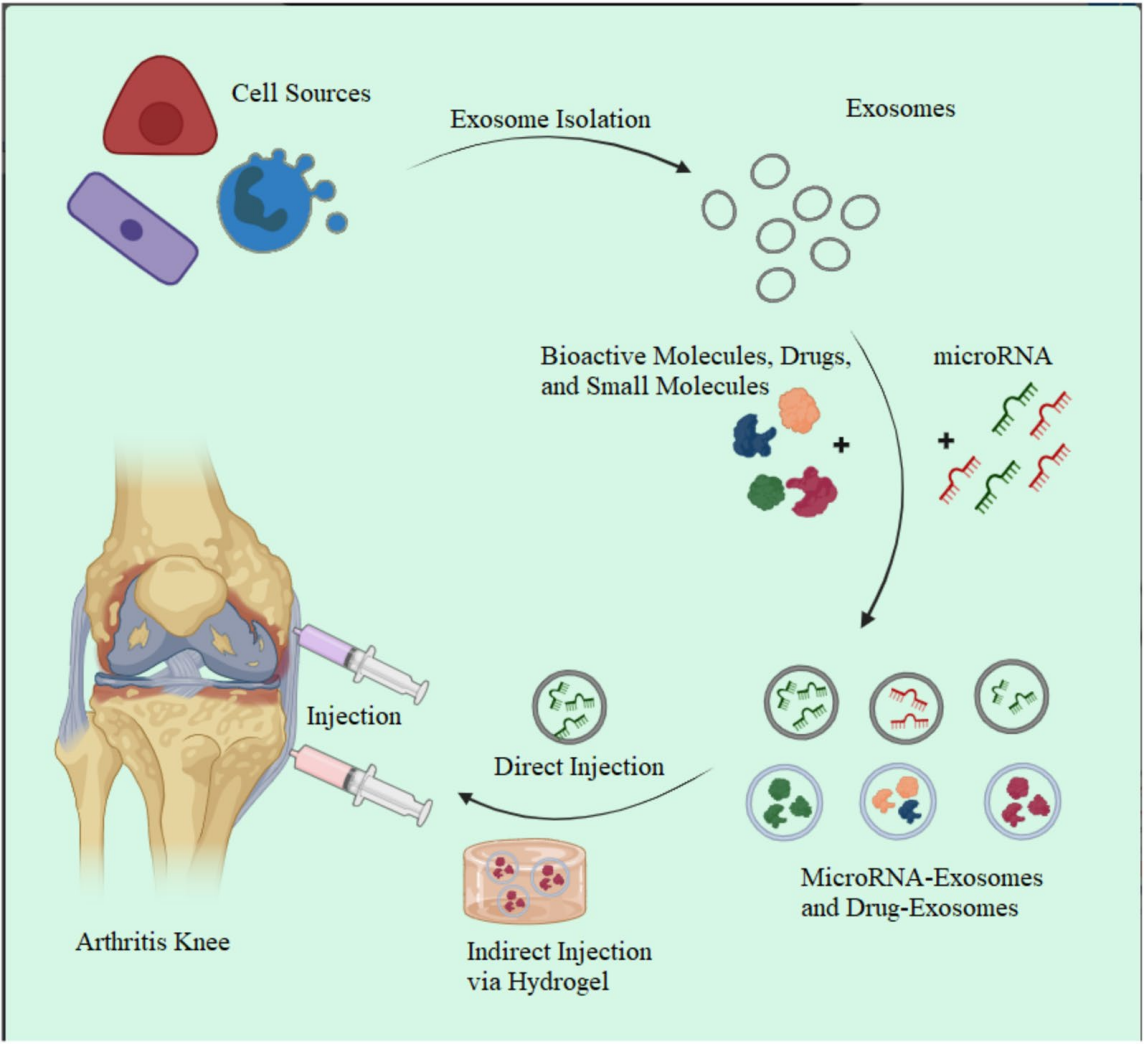
Schematic images illustrate EVs from different sources loaded with different cargoes (microRNA, small molecules, and bioactive), and used for targeted therapy for OA disease. (The figure Created by biorender.com)

Rheumatoid arthritis (RA) is a prevalent chronic autoimmune disorder characterized by systemic inflammation and joint pathology, affecting around 1% of the global population. Notably, women are disproportionately impacted, being three times more susceptible than men. The disease manifests through synovial inflammation, autoantibody generation, and progressive bone and cartilage erosion, culminating in joint deformities and functional impairment, ultimately compromising patients' quality of life. The pathogenesis of RA involves the interaction of various immune cells that secrete various proinflammatory and anti-inflammatory agents, affecting the patient’s synovial tissue and joints [1, 2]. Current therapeutic regimens for RA encompass nonsteroidal anti-inflammatory drugs (NSAIDs), glucocorticoids (GCs), nonbiological disease-modifying anti-rheumatic drugs (DMARDs), and biologic DMARDs (bDMARDs). However, prolonged usage often incurs adverse effects, ranging from gastrointestinal complications to heightened susceptibility to infections, alongside limited efficacy in a subset of patients, coupled with substantial financial burdens [3]. Recently, novel strategies, such as cell and extracellular vesicle-based therapy, have emerged as promising therapeutic approaches for various diseases, including RA. Extracellular vesicles (EVs) are lipid bilayer-bound structures secreted by almost all cell types, including immune cells, and carry various bioactive molecules, such as proteins, lipids, and nucleic acids. They play a crucial role in intercellular communication, immune regulation, and inflammation. In RA, EVs have been shown to have both proinflammatory and anti-inflammatory effects depending on their cellular source and cargo [4, 5]. Despite the promising therapeutic potential of EV-based therapy for RA, several challenges remain, including optimizing the isolation and characterization of EVs, determining the optimal cellular source and cargo for targeted therapy, and ensuring the safety and efficacy of the therapy in clinical settings [6]. In this study, we review new therapeutic advances in RA and discuss the perspectives and challenges of EV-based therapy for RA patients by focusing on selecting the appropriate cellular source of EVs for targeted therapy (Fig. 1).

## Pathophysiology of RA

The pathogenesis of RA is complex and poorly understood. The exposure of susceptible individuals to specific environmental factors causes them to lose self-tolerance. Moreover, autoantibodies, such as rheumatoid factor (RF) and anti-citrullinated protein antibodies (ACPAs), can lead to clinical diseases [7, 8]. Several enzymes, known as peptidyl arginine deiminases (PADs), can cause citrullination in various cell types and tissues. PAD enzymes catalyze the conversion of arginine residues into citrulline [7–9]. Various citrullinated proteins, such as fibrinogen, vimentin, enolase, and type II collagen, are targeted by antibodies against citrullinated peptides, and RF autoantibodies target the Fc region of immunoglobulin G (IgG) proteins. These processes are likely to occur in the lungs, periodontal tissue, joints, and bone marrow [10, 11].

Certainly, in the early stages of this disease, the loss of immune tolerance to endogenous citrullinated antigens results in the onset of anti-cyclic citrullinated peptide (anti-CCP) RA. Osteoclasts, osteoblasts, chondrocytes, and synovial fibroblasts (SFs) are among the resident cells of joints that play a significant role in the pathogenesis of RA together with the immune system. Regarding SFs, evidence suggests that RA development may begin with synovial stromal activation. In a healthy joint, SFs are crucial for preserving joint stability. However, in the context of RA, the activation of these proteins deviates from their natural physiological role due to a range of soluble factors and interactions at the cellular surface. In RA, the subintimal region becomes severely infiltrated with inflammatory cells, such as T and B lymphocytes, macrophages, mast cells, and mononuclear cells, eventually leading to multinucleated osteoclasts. Massive cellular infiltration and new blood vessel development lead to pannus formation [12, 13]. The pannus, which forms at the interface between cartilage and bone, is the primary cause of bone erosion [14].

Secreted cytokines stimulate synoviocytes and rheumatoid arthritis synovial fibroblasts (RASFs), which create enormous amounts of the serine protease cathepsin and matrix metalloproteinases (MMPs)*,* which breakdown the extracellular matrix [15, 16]. MMPs are secreted into the synovial fluid, leading to cartilage breakdown in RA [17]. In addition, cytokine stimulation prompts chondrocytes to immediately release more MMPs into the cartilage [18]. Osteoclasts, the primary agents of bone deterioration, are polarized on bone and populate the synovial membranes of patients with RA [19]. In the presence of macrophage colony-stimulating factor (MCSF), the binding of receptor activator of nuclear factor-κB ligand (RANKL) to its receptor RANK–osteoprotegerin (OPG) on osteoclast precursors promotes osteoclast differentiation [14].

**Fig. 2:**
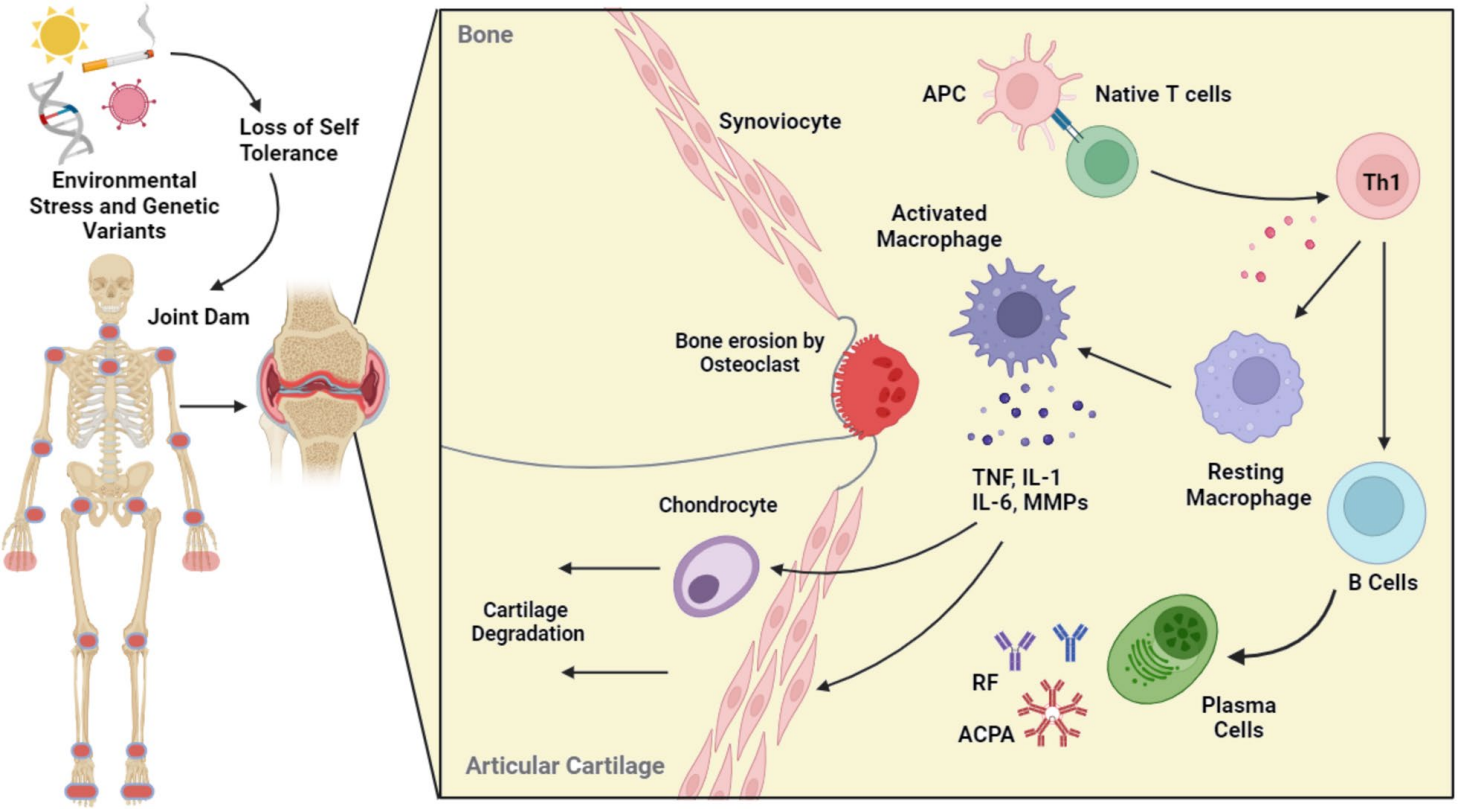
Pathophysiology of Rheumatoid Arthritis. **A** Genetic variants and environmental factors illustrated in the image cause several reactions eventually that promote loss of self-tolerance and subsequently, the inflammatory response of innate, adaptive, and stromal cells causes swelling, cartilage, and bone erosion in certain parts of the human body. **B** Antigen presentation stimulates naive T-cells, especially Th1 cells, and starts an immune response. Subsequently, macrophage activity increases in the synovial joint, which raises the production of pro-inflammatory cytokines such as TNF-α and IL1. These cytokines affect fibroblasts, osteoclasts, and chondrocytes. Matrix-metalloproteinase (MMP) and other collagen-degrading enzymes are released by chondrocytes. Furthermore, T-cells activate plasma cells and B-cells, which lead to secrete a variety of auto-antibodies. These auto-antibodies can attach to APCs and result in pannus formation and subsequent cartilage degradation. (The figure Created by biorender.com)

Furthermore, osteoclastogenesis may be directly initiated by proinflammatory cytokines, including interleukin-1 (IL-1), IL-6, IL-11, and Tumor necrosis factor alpha (TNF-α), and bone resorption occurs through modulating the ratio of RANKL to OPG [20]. The activation of osteoclasts eventually leads to demineralization and corrosion of the bones. Immune cells infiltrate the joint cavity because of pannus formation and inflammatory macrophages subsequently release collagenases, neutral proteases, and proteolytic enzymes that break down cartilage, damaging and destroying it (Fig. 2).

## Regeneration approaches for RA

### Cell/stem cell therapies for RA

In the last two decades, the number of clinical trials of mesenchymal stem cells (MSCs)-based treatment for RA has increased linearly, and promising results have been obtained. The majority of these studies employed allogeneic MSCs due to the immunogenicity of MSCs, their easy availability via cell bank development, and their lower cost than autologous cells [21–24]. The main cell sources are bone marrow and umbilical cord-derived MSCs [25]. In previous studies, no negative effects were recorded at the highest MSC dosage (8 × 108 MSCs/patient), indicating that a variety of MSC dosages might be tolerated [25]; however, scientists have indicated that in terms of short- or long-term efficacy, cell dosages of approximately 1–2 × 106 cells/kg of body weight might be beneficial [21, 26]. Despite advances and considerable variability in ongoing clinical studies of RA treatment involving MSC-based therapy, the ideal approach for determining the MHC context, tissue source, and cell dose is still debated. Various factors are crucial for enhancing the comparability of results in clinical MSC-based studies for RA, such as enhanced uniformity in the standardization of procedures related to MSC treatments and encompassing aspects such as manufacturing processes, MSC sources, MHC contexts, delivery methods, cell quantities, and comprehensive data analysis. Table S1 summarizes the available cell-based therapy studies for RA.

### Gene therapies for RA

The autoimmune nature of RA has made current treatment approaches, such as conventional medications and cell therapy, challenging over the years. Consequently, novel approaches are being developed to overcome these limitations [27]. Gene therapies, as one of these therapeutic strategies, can treat a variety of diseases, such as RA, using genetic engineering techniques [28]. The emerging therapeutic approach for RA aims to either suppress proinflammatory cytokines or enhance the expression of anti-inflammatory cytokines [29, 30]. This intracellular delivery of complementary DNA or RNA as a drug can be achieved through two distinct methods: local transmission (in vivo) and systemic transmission (ex vivo). Although there is an abundance of preclinical trials showing that gene therapy is effective at treating RA experimental models in the library, only a handful of clinical trials have been conducted, confirming its safety and feasibility, with just three protocols progressing to Phase II. As a result, there is currently no conclusive evidence of its efficacy in treating human disease. Safety is the primary concern due to the nonlethal nature of the disease and its impact on life expectancy. A few gene therapy clinical trials for RA are listed in Table S2.

### Tissue engineering for RA

Recently, tissue engineering (TE), a biological substitute that restores, maintains, or improves tissue function, has been used in many diseases, such as osteoarthritis and heart disease [31]. In many cases, TEs seek to regenerate locally damaged tissues or whole organs [31, 32]. The quality of the tissue generated during 3D culture is affected by the type of nutrient input, morphogen stem cells, or precursor cells [32]. However, due to the long duration of current RA treatments, tissue engineering techniques may provide new therapeutic alternatives. Although RA is a systemic disease, several tissue-engineered approaches are currently being evaluated in preclinical phases, but many existing challenges result in limited clinical trials [32, 33]. Some clinical trials of TE for the treatment of RA are listed in Table S3.

### Cell products

The adoption of stem or stromal cell-based therapy is rapidly expanding as a promising therapeutic option for patients with RA who exhibit poor responsiveness or have limited tolerance to existing treatment modalities. Cell-based products such as conditioned medium (CM-MSCs) and platelet-rich plasma (PRP) are believed to play crucial roles in the treatment of diseases such as RA [34].

CM-MSCs are specialized cellular growth environments that are modified via the paracrine effects of MSCs. The production of CM-MSCs entails the cultivation of MSCs in a chemically specified culture medium, such as low-glucose DMEM, until they reach a specific cell population density. Subsequently, the CM-MSCs were isolated, subjected to centrifugation for the elimination of cellular remnants, and preserved at − 80°C for future use [35].

Several growth factors, cytokines, and EVs are secreted by MSCs. These factors are pivotal in the process of tissue regeneration and possess immunomodulatory properties capable of mitigating the intensified pathological immune response observed in patients with RA [35, 36]. CM-MSCs can serve as a cell-free therapeutic approach for addressing diverse pathological states within living organisms, encompassing conditions such as RA [37, 38]. For instance, in a study conducted by Kay et al. [39] CM-MSCs were employed as a cellular substitute in an antigen-induced model of arthritis (AIA). CD4 + T cells derived from the spleens and lymph nodes of arthritic mice treated with CM-MSCs or MSCs were cultured. As a result, CM-MSC or MSC treatment increased the IL-10 concentration and the FOXP3 and IL-4 expression levels and positively affected the regulatory T cells (Tregs) /T helper17 (Th17) balance in the cultured cells. Moreover, CM-MSC therapy diminishes cartilage degradation and exerts inhibitory effects on immune responses. In conclusion, CM-MSCs may be an effective cell-free therapy for inflammatory arthritis, but further analysis is needed.

Platelet-rich plasma (PRP) is an enriched suspension of platelets sourced from the patient’s blood that comprises growth factors and various bioactive compounds. PRP has been extensively utilized for tissue regeneration and pain management, but its application in treating RA has been limited. Only eleven studies were conducted—two in vitro studies, five animal studies, one case report, two case series, and one randomized controlled trial. However, the majority of these studies have reported positive outcomes, such as reduced pain and inflammation, improved function, and no significant adverse effects. The use of medications such as nonsteroidal anti-inflammatory drugs by most RA patients can potentially interfere with the effectiveness of PRP and the diverse methods used in studies. Therefore, additional clinical trials are required to ascertain the safety and effectiveness of cell products as treatments for RA [40]. Furthermore, ongoing clinical trials are exploring the safety and efficacy of EVs for treating RA, offering hope for new therapeutic options for patients with this debilitating disease.

## Extracellular vesicles (EVs) and RA

### Biogenesis and structure

**Fig. 3:**
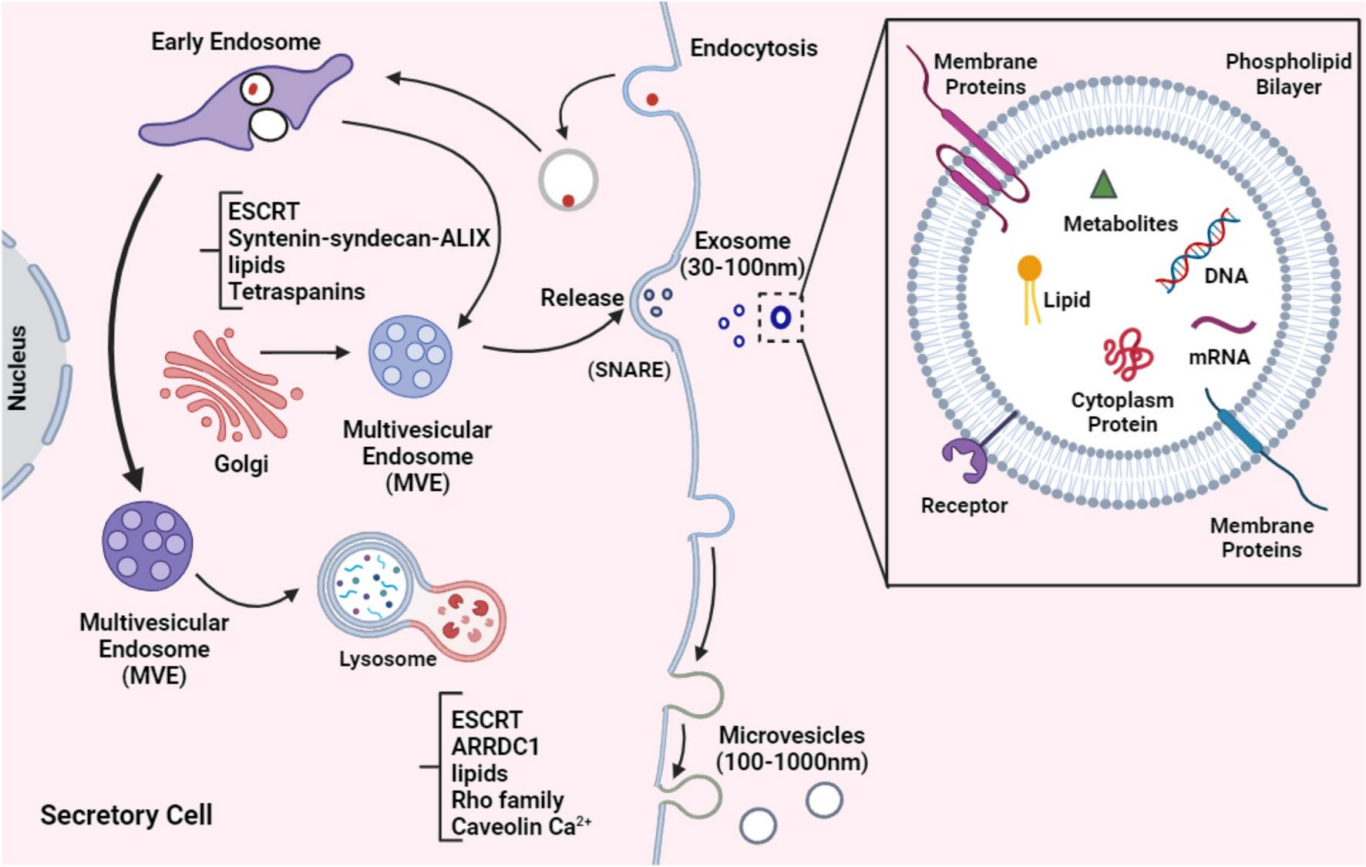
Biogenesis and structure of EVs. **A** Exosomes released by 3 stages: Early endosomes are formed by the inward budding of the plasma membrane, or in some cases originate from the *trans*-Golgi network and generate MVEs. Exosomes are subsequently released upon the fusion of multi-vesicular bodies with the plasma membrane. Alternatively, MVEs can also fuse with lysosomes to be degraded. Several molecules are involved in the biogenesis (e.g., ESCRTs, Syndecan, Tetraspanins, etc.) and fusion of MVEs with the plasma membrane (e.g., SNAREs). Several molecules are involved in the biogenesis and release of macrovesicles. (ESCRTs, ARRDC1, Caveolin Ca2 +). **B** The exosome's composition (protein, lipid, and nucleic acid families) is depicted schematically. It should be noted that each listed component may be present in some EV subtypes but not in others. (The figure Created by biorender.com)

EVs encompass a diverse array of membranous structures released by various cell types and are unable to self-replicate. They are commonly categorized into three groups according to their size and biogenesis: exosomes (30–200 nm), microvesicles (MVs) (100–1000 nm), and apoptotic bodies (> 1000 nm). These cells also express the CD63, CD81, and CD9 biomarkers [41]. Exosome formation commences through a process initiated by the double inward folding of the plasma membrane. The initial inward folding results in the formation of vesicles with a cup-like structure, ultimately progressing into late-sorting endosomes. The endoplasmic reticulum membrane plays a role in subsequent inward folding, leading to the development of intracellular multivesicular bodies housing intraluminal vesicles. These vesicles merge with the plasma membrane and are ultimately discharged as the final exosome during exocytosis (Fig. 3) [5]. In terms of structure, EVs contain abundant cargo, including nucleic acids, proteins, lipids, etc. Notably, the amount of each substance in EVs varies based on the origin cell type. EV-RNA is often shorter than ordinary cellular EV-DNA, the sizes of which range from 100 bp to 2.5 kB. Concerning protein content, various protein types, particularly MHC II, tetraspanins, ESCRT proteins, TSG101, and heat shock proteins, are frequently found in EVs [42].

### Source and protocol for EV production

**Fig. 4:**
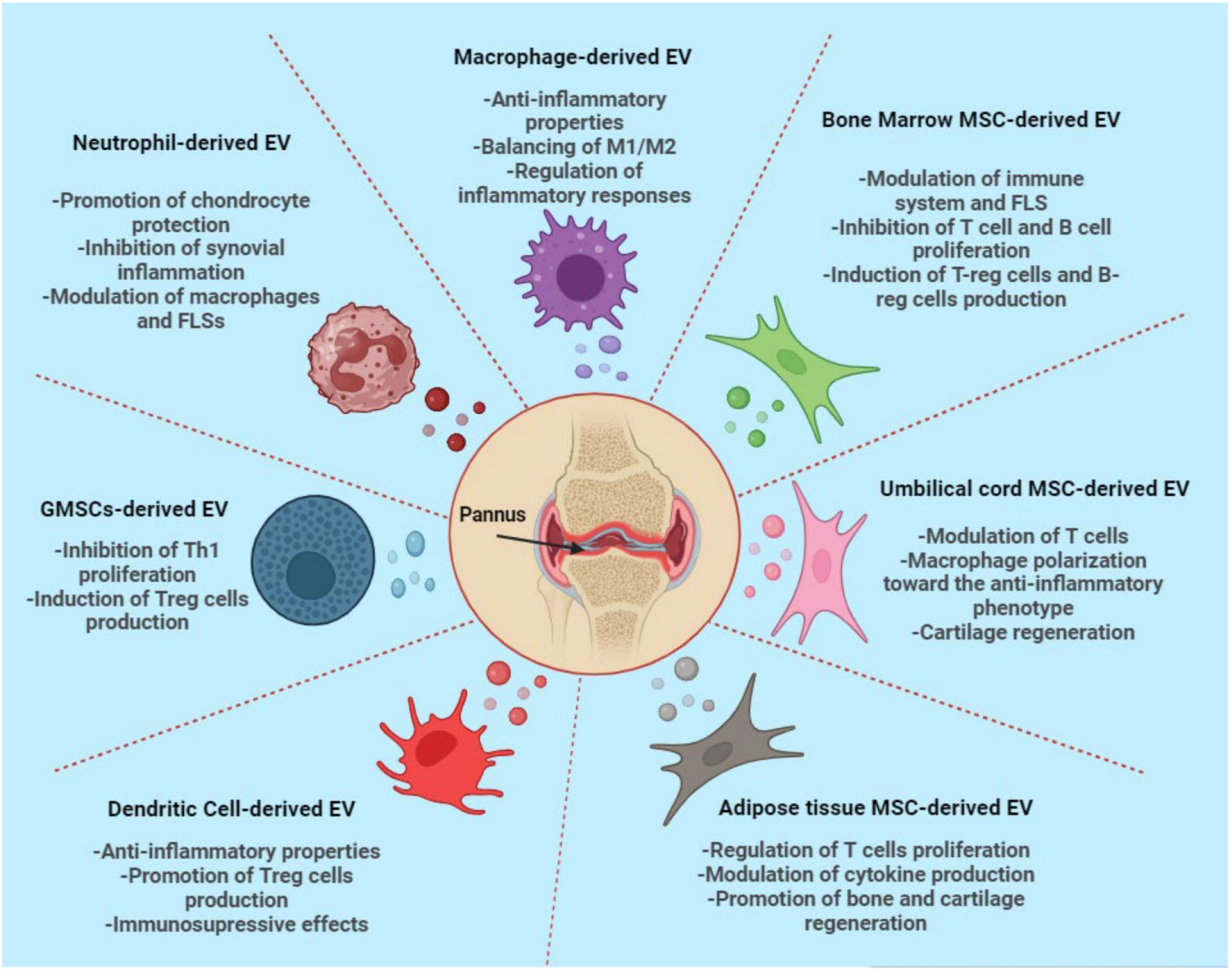
Different cellular sources of EVs in RA treatment. Various cell types, including mesenchymal stem cells (MSCs), dendritic cells, macrophages, neutrophils, and granulocyte myeloid-derived cell-derived suppressor EVs (GMSC-EVs), release specific EV subpopulations that contribute significantly to modulating RA pathogenesis and treatment strategies. These EVs possess potent anti-inflammatory and immune-modulating properties and are essential in preserving the integrity of bone and cartilage. (The figure Created by biorender.com)

As research in the field of EVs progresses, it is crucial to identify the optimal cellular sources of EVs and to standardize isolation and characterization methods to establish a reliable and reproducible protocol. Here, we provide a more detailed explanation of the various cellular sources of EVs, their unique characteristics, and the therapeutic potential of each source (Fig. 4).

#### Mesenchymal stem cell-derived EVs

MSC-EVs are lipid bilayer structures secreted by MSCs in resting or activated states. The immunomodulatory capabilities and tissue regeneration potential of these materials are comparable to those of their parent MSCs [5, 43]. MSC-EVs are perceived to pose a diminished risk of adverse effects, including teratoma formation and immune rejection, compared with viable cells. 36,37. MSC-EVs hold great potential as a therapeutic approach for RA because of their anti-inflammatory and immunomodulatory properties [4]. These EVs have demonstrated the ability to alleviate disease progression and mitigate joint damage in patients with RA [44, 45]. MSC-EVs exert a range of beneficial effects, including the inhibition of T cells, B cells, and dendritic cells (DCs) and macrophage activation and proliferation. Conversely, they promote the expansion of Tregs and myeloid-derived suppressor cells (MDSCs), which contribute to immune regulation. Notably, MSC-EVs suppress the production of proinflammatory cytokines such as TNF-α, IL-1β, and IL-6 while enhancing the secretion of anti-inflammatory cytokines such as IL-10 and transforming growth factor-beta (TGF-β). Furthermore, MSC-EVs have been shown to stimulate the differentiation of chondrocytes and osteoblasts, thereby promoting the regeneration of bone and cartilage [5, 45–47].

Among the various sources of MSCs, extensive research has focused on bone marrow MSCs (BMSCs) for their potential in RA therapy. Moreover, BMSCs have been demonstrated to be effective at alleviating symptoms and exerting immunomodulatory effects in patients with refractory RA [48]. Similarly, BMSC-derived EVs (BMSC-EVs), which share therapeutic effects with their parent cells, have exhibited notable effectiveness in alleviating experimental RA. The underlying mechanism involves the modulation of immune cells and fibroblast-like synoviocytes (FLSs), which are key contributors to RA pathogenesis [49, 50]. Furthermore, BMSC-EVs have been empirically shown to alleviate RA by suppressing the proliferation of T and B lymphocytes while also inducing the dose-dependent differentiation of Tregs and regulatory B cells expressing IL-10 [51]. In addition, they suppress the activation, migration, and invasion of FLSs by transferring specific microRNAs (miRNAs) that promote signaling pathways involved in inflammation, proliferation, and angiogenesis [52–54]. Moreover, BMSC-EVs facilitate bone and cartilage regeneration as well as angiogenesis by delivering growth factors and bioactive molecules [55, 56].

Adipose tissue-derived MSCs (AMSCs) are another promising source of EVs for RA therapy, as evidenced by the research conducted by Gonzalez-Rey et al. Their study highlighted the significance of AMSCs in regulating the proliferation of collagen-reactive T cells and cytokine production and underscored their pivotal role in contributing to the improvement of both RA symptoms and disease activity [57]. Furthermore, Bolandi et al. demonstrated that AMSC-derived EVs effectively modulate immune cells and FLSs by inhibiting proinflammatory subsets of CD4 + T cells and inducing anti-inflammatory subsets through the transfer of specific miRNAs [58]. Additionally, AMSC-EVs promote cartilage and bone regeneration by delivering growth factors and bioactive molecules [59]. Importantly, AMSC-EVs offer advantages such as easy accessibility and a greater association with proteins related to immunomodulation, positioning them as appropriate cellular sources of EVs in RA therapy [59].

The investigation of umbilical cord MSCs (UCMSCs) as a potential therapeutic option for RA has yielded promising findings. Miranda et al. isolated UCMSC-derived EVs in a three-dimensional culture and demonstrated enhanced efficacy, primarily attributed to the increased production of exosomes [60]. These EVs can ameliorate collagen-induced arthritis by modulating T lymphocytes and restoring the balance between proinflammatory and anti-inflammatory factors [61]. Moreover, their contribution to cartilage regeneration is evidenced by the delivery of growth factors and miRNAs to chondrocytes and macrophages, thereby promoting chondrocyte proliferation and inducing macrophage polarization toward the anti-inflammatory and pro-regenerative M2 phenotype [62].

A significant challenge in using MSC-derived EVs for therapeutic purposes is their limited proliferative potential. As MSCs are expanded in vitro, their biological properties decline, leading to less efficacious EVs from late-passage MSCs [63, 64]. This decline is due to cellular senescence and reduced stem cell-like qualities, which affect the therapeutic efficacy of the bioactive molecules in the EVs [65, 66]. To address these challenges, immortalized MSCs (iMSCs) and iPSC-derived MSCs (iEVs) offer promising solutions. iMSCs provide a stable and scalable source for producing therapeutic EVs by suppressing p53- and Rb-mediated pathways and preserving telomeres through transfection with immortal genes such as SV40 large T antigen (SV40LT), human papillomavirus E6/E7, or human telomerase reverse transcriptase (hTERT) [67–69]. Research has shown that EVs from iMSCs can enhance therapeutic efficacy in RA models by increasing anti-inflammatory cytokines and reducing pro-inflammatory cytokines, thereby alleviating cartilage damage [70]. Similarly, current research indicates that iPSC-derived MSCs-derived EVs (iEVs) are a promising alternative to tissue-derived MSCs for therapeutic applications, especially in immunomodulation and treating immune-mediated diseases [71–73]. These EVs have comparable therapeutic efficacy to their parent iMSCs, effectively alleviating conditions like secondary Sjögren's syndrome in NOD mice by inhibiting lymphocyte infiltration and reducing autoantibody levels [74, 75]. Therefore, the use of these EVs can be considered in the treatment of RA. These attributes make both iMSCs and iEVs valuable sources of EVs for the treatment of RA.

Although MSC-EVs have demonstrated potential in both in vitro and animal models of RA, it is crucial to consider that preclinical models cannot fully replicate the complexities of this disease. Consequently, it is imperative to avoid misinterpreting the suppression of experimental arthritis as an advantageous outcome in RA patients. Although clinical trials are presently in progress to assess the safety and effectiveness of MSC-EVs in addressing different inflammatory disorders and osteoarthritis, there have been no documented clinical trials exclusively dedicated to treating RA. Conversely, before clinical translation, additional meticulously planned preclinical investigations should be conducted to explore elements such as ideal dosage levels, delivery methods, treatment schedules, enduring consequences, and potential negative outcomes [45].

#### Neutrophil-derived EVs

Neutrophil-derived EVs are highly present in the synovial fluid of patients with RA. These EVs play a crucial role in promoting chondroprotective effects and exhibit anti-inflammatory properties by inhibiting synovial inflammation and modulating macrophages and FLSs in the joint [76]. Researchers have specifically identified neutrophil-derived AnxA1 + MVs isolated from the synovial fluid of patients with RA as powerful mediators. These MVs increase the production of TGF-β by chondrocytes, leading to enhanced extracellular matrix accumulation and reduced cartilage destruction in a mouse model of arthritis (K/BxN) [77]. Notably, these MVs demonstrate selectivity in modulating macrophage polarization, reducing classical activation, and promoting the release of TGF-β. The effect of phosphatidylserine on MVs plays a pivotal role in regulating macrophage polarization markers. Furthermore, the binding of annexin-A1 and its receptor, formyl-peptide receptor type 2, to MVs significantly influences the macrophage response [78].

Importantly, the impact of neutrophil MVs extends beyond macrophages and affects the behavior of FLSs in coculture settings, highlighting their influence on the overall inflamed microenvironment. Rhys et al. used a macrophage/FLS coculture system to demonstrate the therapeutic potential of vesicles for crosstalk between these cells. They also used a murine arthritis model and showed that the therapeutic potential of neutrophil-derived MVs is due to their ability to induce a switch in the macrophage phenotype within inflamed joints [78]. Thomas et al. also showed that in murine inflammatory arthritis, neutrophil-derived EVs reduce the loss of sulfated glycosaminoglycans and protect against IL-1-induced cartilage breakdown. They also induce an anti-inflammatory macrophage phenotype characterized by decreased MHCII and CD86 expression and increased CD206 expression [79]. Zhang et al. demonstrated that neutrophil-derived exosomes functionalized with ultrasmall Prussian blue nanoparticles (uPB-Exos) have promising outcomes in targeting inflamed tissues and improving joint damage in a CIA mouse model by regulating Th17/Treg cells and neutralizing proinflammatory factors [80]. The therapeutic potential of neutrophil-derived EVs in treating RA offers promising avenues for managing this disease because of their ability to protect against cartilage, modulate inflammation, and induce beneficial changes in immune cell behavior. Nevertheless, since these EVs mirror the characteristics of their parent cells, it is essential to remove bioactive compounds without beneficial effects to prevent increased inflammation and minimize adverse effects. Furthermore, ongoing research is needed to improve the efficacy of neutrophil-derived EVs at specific target sites, ensuring optimal pharmacokinetics and minimal side effects. Finally, advancing the findings from both in vitro and in vivo studies to clinical trials is imperative.

#### Granulocytic myeloid-derived suppressor cell-derived EVs (GMSC-EVs)

Myeloid-derived suppressor cells (MDSCs) represent a highly diverse population of immature cells originating from the bone marrow and are critical for immunosuppression under autoimmune conditions [81]. Two main subsets of MDSCs, monocytic-MDSCs (M-MDSCs) and granulocyte-MDSCs (G-MDSCs), exhibit distinct suppressive functions. M-MDSCs suppress CD4 + T-cell proliferation, whereas G-MDSCs inhibit T-cell function by producing reactive oxygen species (ROS). These suppressive effects of MDSCs result in the inhibition of CD4 + T-cell proliferation and the promotion of proinflammatory Th17 cells. MDSCs also suppress cytokine production by CD4 + T cells and inhibit B-cell proliferation activation and antibody production. Studies involving MDSC transfer or suppression in animal models and patients with RA have shown positive outcomes, including reduced arthritis severity, decreased numbers of Th17 and CD4 + T cells, and decreased levels of proinflammatory cytokines in joint tissues and plasma [81, 82]. The anti-inflammatory effects of MDSC-derived EVs have been investigated in various studies. For instance, Wang et al. showed that G-MDSC-derived exosomes possess arginase-1 (Arg-1) activity, which plays a crucial role in the immunosuppressive function of G-MDSCs in dextran sulfate sodium (DSS)-induced colitis by inhibiting the proliferation of Th1 cells and promoting the expansion of Tregs [83]. These findings indicate that G-MDSC-derived exosomes share some biological functions with their parent cells.

MDSC-derived EVs have shown potential as therapeutic mediators for treating RA. In another study using a murine RA model, Zhu et al. demonstrated that exosomes derived from G-MDSCs have notable efficacy in alleviating arthritis and reducing the proportions of Th1 and Th17 cells. Further investigation revealed that specific miRNAs, including miR-29a-3p and miR-93-5p, present in these exosomes targeted key molecules such as T-bet and STAT3, resulting in the suppression of Th1 and Th17 cell differentiation [84].

In a recent study by Wu et al., G-MDSC-derived EVs were shown to contain high levels of prostaglandin E2 (PGE 2), which plays a crucial role in promoting the generation of regulatory B cells (Bregs) with immunosuppressive functions. This research demonstrated that the administration of G-MDSCs has a beneficial impact on joint damage, decelerates the progression of this disease, and decreases antibody concentrations in mice afflicted with CIA. Furthermore, G-MDSC-derived EVs influenced the frequency of plasma cells and T follicular helper cells (Tfh cells) and upregulated the proportion of B cells producing interleukin-10 (IL-10). The mechanism underlying the effect of G-MDSC-derived EVs involved activation of the GSK-3β pathway and phosphorylation of GSK-3β and cAMP response element-binding protein (CREB) in B cells [85]. The use of G-MDSC-derived EVs for treating RA is a promising new approach, but some challenges must be overcome. One of the biggest challenges is the variability in EV production owing to differences in subpopulations, microenvironments, and genetic backgrounds across studies [84]. This makes it imperative to achieve a standardized and consistent source of drugs to ensure reliable therapeutic outcomes. The cargo of G-MDSC-derived EVs is a complex mixture of various proteins, mRNAs, and miRNAs, and identifying their precise role in immunomodulation is challenging. Elucidation of the specific functions of each component of cargo is essential for accurate elucidation of therapeutic mechanisms. Additionally, the variability of G-MDSC-derived EV functions at different stages of RA adds complexity to therapeutic interventions and necessitates a tailored approach to disease progression. To successfully develop and apply G-MDSC-derived EVs in RA therapy, ongoing research, and technological advancements must overcome these challenges.

#### Dendritic cell-derived EVs

Dendritic cells (DCs) serve as vital mediators of innate and adaptive immune responses and play crucial roles in coordinating and regulating immune reactions. The maturation status of DCs is a key determinant in shaping the balance between immune tolerance and immune activation, highlighting the dynamic nature of DC-mediated immunoregulation [86, 87]. DC-derived EVs have garnered significant interest as cell-free therapeutic agents for treating inflammatory diseases. These DC-derived EVs mimic the biology of donor DCs and play pivotal roles in immune regulation and activation. DCs generate two types of EVs, mature DC-derived EVs (mDC-EVs) and immature DC-derived EVs (imDC-EVs), each of which have unique properties and potential therapeutic applications. In this regard, del Cacho et al. showed that mDC-EVs exhibit immune-activating properties, leading to successful tumor eradication and pathogen elimination in vitro and a Chicken model [88], while immature or tolerogenic DC-derived EVs can induce immune tolerance, making them potentially valuable in transplantation and autoimmune disease scenarios. Notably, in animal models, the administration of immature DC-derived EVs resulted in prolonged survival among transplant recipients. Additionally, these EVs led to a reduction in the clinical symptoms observed in mice afflicted with autoimmune diseases [89–92]. Previous studies have shown that modifying EVs derived from DCs with immunomodulatory molecules has beneficial effects on reducing the severity of RA in murine CIA and suppressing inflammation in a murine delayed-type hypersensitivity (DTH) model. The involvement of key molecules such as MHC II, FasL, IDO1, B7-1/2, IL-10, and IL-4 contributes to the immunosuppressive and anti-inflammatory properties of these EVs [90, 93, 94]. To further enhance the therapeutic potential of DC-derived EVs, researchers have explored surface engineering techniques. One such technique is the use of reactive oxygen species (ROS) responsive tolerogenic DC-derived exosomes (TolDex), which have been proven to be effective at treating RA. In this regard, Lee et al. reported that TolDex surface engineering significantly decreased IL-6 and CD40 levels while promoting the production of regulatory T cells [91]. While there is substantial evidence highlighting the immunomodulating functions of DC-derived EVs, much of the related research has been conducted using in vitro-differentiated DCs. Limited information exists regarding the functionality of these vesicles when released by DCs in vivo, necessitating further research to understand their in vivo functionality, particularly under inflammatory conditions. Understanding the pathways involved in the biogenesis of these EVs is crucial, but challenges arise due to their low abundance, which requires a substantial number of secreting cells for analysis. Developing methodologies that enable the study of EVs on a small scale is essential for revealing their therapeutic potential in future clinical investigations.

#### Macrophage-derived EVs

Given their abundance in synovial tissue and their association with disease severity, macrophages are pivotal for treating RA. These versatile cells can switch between proinflammatory M1 and anti-inflammatory M2 phenotypes based on local signals, and maintaining a balanced M1/M2 ratio is crucial for therapeutic success in RA. Macrophages communicate with target cells through direct contact and secretion of cytokines and EVs, which influence immune responses in inflammatory diseases. Specifically, macrophage-derived EVs have been found to play a crucial role in regulating inflammatory responses [95, 96]. M2 macrophage-derived EVs possess anti-inflammatory properties and have the potential for targeted delivery of anti-inflammatory drugs [97]. These EVs can effectively re-establish M1–M2 macrophage equilibrium in RA synovial tissue, thereby ameliorating synovial inflammation and protecting against joint destruction [98]. For instance, Zhang et al. showed that modified M2 macrophage-derived EVs have impressive inflammation-targeting capabilities, suggesting that they could be potential treatments for inflammatory conditions such as RA and spinal cord injury (SCI) [99].

Previous studies have explored the potential of M2 macrophage-derived EVs as a drug delivery system for RA treatment. These EVs, engineered with cell-penetrating peptides and loaded with therapeutic compounds, have shown promising effects on macrophage polarization, promoting repolarization to the anti-inflammatory M2 type. In a recent study by Li et al. in mouse models of RA, cell-penetrating peptide-modified primary M2 macrophage-derived exosomes were shown to significantly reduce swelling, inhibit bone destruction, and improve functional recovery in comparison with those in the control group (treated with EVs without modification) [100]. Furthermore, the use of FA-PEG-Chol (FPC) to modify macrophage-derived EVs has enhanced the targeting ability of these cells, leading to sustained drug release and significant reductions in the inflammatory response and bone degradation in animal models of RA [101]. These findings highlight the considerable potential of macrophage-derived EVs as a promising approach for drug delivery and therapeutic intervention in the treatment of RA. Additionally, a novel strategy utilizing macrophage-derived EV (MEV)-coated nanoparticles (MNPs) has shown promise in the targeted delivery of therapeutics to sites of RA. Li et al. showed that encapsulating the drug tacrolimus within MNPs effectively suppressed the progression of RA in mice, highlighting the potential of this approach for RA treatment [102].

In another innovative approach, researchers have created inherent anti-inflammatory EVs (AI-EVs) by integrating macrophage-derived exosomes with the anti-inflammatory immune modulator interleukin-10 (IL-10). Noninvasive ultrasound was used to enhance the targeted accumulation of AI-EVs in inflammatory tissues. This study demonstrated that ultrasound-augmented AI-EVs promote macrophage polarization to the M2 phenotype, diminish signs of inflammation, stimulate resolution, and expedite tissue restoration in CIA [103]. These findings suggest significant targeted anti-inflammatory therapeutic effects and provide insights for the treatment of RA and other inflammatory diseases. Furthermore, the development of hybrid EV-mimicking nanovesicles (HNVs) through the fusion of an M1 macrophage membrane with EV-mimicking nanovesicles derived from M2 macrophages provides a comprehensive anti-inflammatory effect. Zhao et al. presented black phosphorus nanosheets (BPs) to HNV (HNV@BP) to reduce inflammation upon near-infrared (NIR) irradiation. They also showed that HNV@BP effectively targets and suppresses inflammation in a mouse model of CIA [104].

In conclusion, macrophage-derived EVs hold immense therapeutic potential in managing inflammatory diseases, particularly in the treatment of rheumatoid arthritis. These EVs can effectively modulate macrophage polarization, restore the M1–M2 equilibrium, and ameliorate synovial inflammation. The inflammation-targeting capabilities, drug delivery potential, and ability to enhance tissue repair of these cells make them promising candidates for future therapeutic interventions. However, the use of these compounds for therapeutic purposes faces several challenges, particularly in regulating their release and content to prevent disease development and progression. Moreover, macrophage-derived EVs may not replicate the diverse and immediate responses of macrophages to different environments, which necessitates their precise modulation based on specific diseases or conditions. While reprogramming macrophage-derived EVs shows promise, traditional isolation methods yield limited quantities and functionality, which necessitates the development of more efficient techniques. Addressing unresolved issues, such as isolation and purification efficiency, is critical for advancing the applications of macrophage-derived EVs. Nevertheless, characterizing distinct subtypes of macrophage-derived EVs based on molecular markers remains a challenge. Therefore, further research is crucial for understanding the mechanisms of macrophage-derived EVs and addressing these challenges, paving the way for effective therapeutic strategies in the future, especially in the context of RA. All clinical and preclinical studies related to EV therapy for RA are summarized in Table 1.

**Table 1:** The list of studies related to EV therapy of RA

Source	Subtype	Size	Isolation method	Characterization	Outcome	References
BMSCs	Exo	100 nm	Total Exosome Isolation Reagent (Invitrogen 4,478,359)	Size and Zeta potential: DLS; morphology: TEM; surface markers: Western blot and flow cytometry	Suppression of proliferation and migration of synoviocyte fibroblast-like cell line in virto Inducing apoptosis of FLS cell line in virto	[15]
BMSCs	Exo	120 nm	0.22-μm filtration and ultracentrifugation	Size: NTA; morphology: TEM; surface markers: Western blot	Inhibition of proliferation, motility, and inflammation in RA-FLS Inducing apoptosis of RA-FLS cells by inactivating the NF-κB pathway	[12]
BMSCs	Exo	40–100 nm	Ultracentrifugation	Size: NTA; morphology: TEM; surface markers: Western blot	Suppression of RA-FLS function in virto Reduction of arthritis and bone damage in CIA mice In vivo	[17]
BMSCs	EVs	93 nm	Differential centrifugation	Size: NTA; morphology: TEM; surface markers: Western blot; protein concentration; BCA assay	Inhibition of RA-FLS proliferation and resistance to apoptosis through miR-34a in vitro Reducing RA inflammation In vivo	[87]
BMSCs	Exo	30–100 nm	Differential centrifugation	Size: NTA; morphology: TEM; surface markers: Western blot	Inhibition of survival and increase of apoptosis of RA-FLSs Suppression of inflammation score, joint destruction, and inflammatory response in RA mouse model Direct inhibition of cell apoptosis by suppressing inflammatory cytokines, rheumatoid markers, and immunological markers, and mediating the NF-κB pathway	[13]
BMSCs	Exo	30–100 nm	Ultracentrifugation	Size: DLS; morphology: TEM; Surface marker: western blot	Reduction of mFLS cell proliferation and inflammatory cytokines secretion by delivering miR-21 Alleviation of RA symptoms and joint damage in mice by modulating the miR-21-TET1-KLF4 regulatory axis	[16]
BMSCs	Exo		Exosome Isolation Kit	Size: DLS; morphology: TEM;	Decreased expression and secretion of MMP14 and VEGF in FLS of RA patients Inhibition of migration and invasion of RA FLS and tube formation of HUVECs, by targeting MMP14 and VEGF Decreasing paw thickness and arthritis scores in mice with collagen-induced arthritis Decreasing joint destruction by inhibiting synoviocyte hyperplasia and angiogenesis in mice with collagen-induced arthritis	[18]
AMSCs	Exo	150 nm	Differential centrifugation and 0.22-μm filtration	Size: NTA; morphology: TEM; surface markers: Western blot	stimulation of migration, proliferation, and chondrogenic and osteogenic differentiation of BMSCs in vitro cartilage and bone regeneration in vivo	[21]
AMSCs	MV	218–230 nm	Exosome Isolation Kit (ExoEasy Maxi kit), ultracentrifugation	Size: NTA; morphology: TEM	Reduction of joint swelling and proinflammatory cytokine expression in RA mice	[19]
UCMSCs	MV	40–300 nm	Gradient ultra-high-speed centrifugation	morphology: TEM; protein concentration; BCA assay	Reduction of Th17 cell ratio and IL-17 level and increase of Treg cell ratio and Treg/Th17 ratio and TGF-β level in virto	[24]
UCMSCs	Exo	160 nm	Gradient ultra-high-speed centrifugation	Size: NTA; morphology: TEM; surface markers: Western blot; protein concentration; BCA assay	Decreasing the severity of arthritis and synovial hyperplasia in CIA rats Inhibition of T lymphocyte proliferation and induction of T lymphocyte apoptosis in CIA rats Decreasing the proportion of Th17 cells and increasing the proportion of Treg cells in the spleen of CIA rats Decrease of serum IL-17 and increase of serum IL-10 and TGF-β in CIA rats Decreased RORγt and increased expression of FOXP3 in the spleen and decreased expression of RORγt and FOXP3 in the joints of CIA rats	[88]
Neutrophils	MV	70–400 nm	Differential Centrifugation	Size: NTA; Analysis and counting: ImageStream analysis	Restriction of the ability of macrophages to activate FLSs Inhibition of the macrophage’s activation Induction of TGF-β releasing	[28]
Neutrophils	MV	< 412 nm	Density gradient centrifugation	Size: NTA Analysis and counting: ImageStream analysis	Activating chondrocytes and modulating anti-inflammatory pathways Increasing extracellular matrix production and cartilage protection	[27]
Neutrophils	Exo	116 nm	Differential centrifugation and 0.22-μm filtration	Size and Zeta potential: NTA; morphology: TEM; protein quantification: BCA assay; surface markers: Western blot	Alleviating inflammatory stress in the joints Inducing a cascade of anti-inflammatory events by regulating the balance between Th17 and Treg cells	[30]
G-MDSCs	Exo	30–110 nm	ExoQuick-TC™ Exosome Isolation Kit	Size and morphology: TEM; surface markers: Western blot	Suppressing the differentiation of pro-inflammatory Th1 and Th17 cells in vitro and In vivo Reducing arthritis severity, joint damage, and immune cell infiltration In vivo	[34]
G-MDSCs	Exo	99.6 nm	0.22-μm filtration, centrifugation, and exosome extraction kit	Size: NTA; morphology: TEM; protein concentration: BCA assay; surface markers: Western blot	Increasing IL-10 secretion by splenic B cells, both In vivo and in vitro Reducing arthritis severity and inflammatory cell infiltration In vivo	[35]
DCs	Exo	< 100nm	Differential centrifugation	Size and morphology: TEM; surface markers: Western blot and FACS	Targeting CD8 + effector T cells and activating T regulatory cells Reversing established CIA and reduced inflammation in the DTH footpad model	[41]
DCs	Exo	Not mentioned	Differential centrifugation	morphology: TEM; surface markers: Western blot and FACS	Reduction of swelling in treated and untreated paws in mouse DTH model Amelioration of established CIA in mice	[89]
DCs	Exo	68,169, and166 nm	Differential centrifugation, 0.22-μm filtration, and ultrafiltration (TFF systems)	Size & number: NTA; morphology: TEM; surface markers: Western blot	Regulation of the secretion of pro-inflammatory cytokines in vitro Improvement of accumulation in the joints after intravenous administration In vivo Decreased levels of IL-6, increased TGF-β, and induction of regulatory T cells In vivo	[42]
Macrophages	Exo	124 nm	Differential centrifugation	Size and zeta potential: NTA; morphology: TEM; surface markers: Western blot	Promoting the repolarization of macrophages in vitro Targeted accumulation at inflammation sites Suppression of inflammation and improved motor function in SCI and RA models	[51]
Macrophages	Exo	98.87 ± 6.69 nm	Gradient Centrifugation, ultrafiltration and ultracentrifugation	Size, polydispersity index (PDI), and zeta potential: DLS; morphology: TEM; surface markers: Western blot	Increased endocytosis and anti-inflammatory effect in vitro Increased accumulation in inflamed joints In vivo Reducing the number of inflamed joints and protecting bone and cartilage in CIA rats	[52]
Macrophages	MV	1000 nm	Serial centrifugation 1000 and 4000 rpm	Size and zeta potential: DLS; morphology: fluorescence image; protein content: BCA assay	Strong attachment to inflamed HUVECs in virto Increasing the capacity to target inflammation In vivo Suppressing the progression of RA in CIA mice	[53]

*Exo* Exosomes; *EVs* Extra cellular vesicles; *MV* Macrovesicles

### EV isolation method

Standardizing methods for isolating EVs presents a significant challenge in clinical application. The technique used for isolating EVs has a profound impact on sample yield and purity [105]. EVs, which have effective functions in intercellular communication across different body compartments, can be found in various biofluids. Researchers have developed different isolation techniques and compared their efficiency. These methods can be categorized into four groups: ultracentrifugation (UC), size-based isolation, precipitation, and affinity-based methods. However, a comprehensive worldwide survey of EV isolation and characterization techniques revealed that no universally accepted "gold standard" method currently exists for EV isolation and purification [106].

Historically, UC-based methods have been the most popular for the primary isolation of EVs from cell culture media and biofluids [107]. UC, a simple and cost-effective technique, is widely used for EV isolation. One specific approach, known as differential UC, involves multiple centrifugation steps at varying forces. Initially, low-speed centrifugation at 300 × *g* and 2500 × *g* was performed to eliminate cells and larger debris from the sample. Subsequent centrifugation steps at 10,000 × *g* and 100,000 × *g* or 200,000 × *g* were then carried out to pellet larger and smaller EVs, respectively [108]. However, despite these steps, complete separation is not achieved, and there are drawbacks associated with this method. The high centrifugal forces used in UC can lead to vesicle clumping and the recovery of smaller contaminants. Moreover, UC is time-consuming, has large output variations, and may compromise the structural and biological integrity of EVs [109].

To address these limitations, UC can be combined with density gradient techniques that match the specific density of EVs, which typically range from 1.13 to 1.19 g/ml. Density gradient solutions such as sucrose or iodixanol are commonly used for this purpose. During centrifugation, components with different buoyant densities reach a static position in the layer of medium with similar density, facilitating the removal of most contaminants [110, 111]. While UC with density gradients is valuable for laboratory-based research, its application in clinical settings is limited by its time-intensive preparation, significant equipment requirements, and limited scalability for high-throughput applications [105].

In response to these limitations, alternative size-based separation strategies, such as ultrafiltration and size-exclusion chromatography, have been introduced. These techniques offer simplified and highly efficient exosome isolation and are commercially available as exosome separation kits. Ultrafiltration relies on the size and molecular weight cutoff (MWCO) of a membrane filter, which involves passing EVs in suspension through membrane filters with specific size exclusion thresholds, typically ranging from 0.1 to 0.45 μm pore diameters. Particles larger than the MWCO are retained on the filter, while smaller particles pass through the filter [112]. Ultrafiltration provides a fast and cost-effective method for separating EVs from larger elements, resulting in the preparation of individual EV particles rather than aggregation. However, this approach can lead to EVs with high protein contamination and concerns about its impact on EV integrity. To achieve high purity, ultrafiltration may need to be combined with other techniques, such as UC and size exclusion chromatography (SEC) [112–114].

SEC is a chromatographic technique that utilizes a permeable immobile phase to separate components according to their hydrodynamic radii [115]. It offers preservation of vesicle structure, integrity, and biological activity but has longer run times and limited scalability [116]. Notably, the qEV Exosome Isolation Kit represents an advancement in SEC-based exosome isolation, providing rapid and precise isolation and promoting standardization for clinical applications [117, 118]. Commercial kits for EV isolation have been developed to circumvent the limitations of conventional methods. However, these kits vary in terms of reliability, specificity, and cost-effectiveness, often restricting the analysis to a finite quantity of samples. For example, the ExoMir Kit utilizes membranes with different pore sizes to separate exosomes based on size, discarding the smallest vesicles. Similar methods, such as ExoTIC, have been developed to enhance the clinical applicability of exosome isolation [119.

Precipitation-based EV isolation serves as an alternative to ultracentrifugation and offers certain advantages. Precipitation kits and polymers exploit the changes in the solubility and aggregation behavior of EVs rather than relying on their density and size [120]. The polymeric precipitation method involves the formation of vesicle aggregates by adding water-excluding polymers such as polyethylene glycol (PEG) or lectins to the sample. This process effectively removes larger contaminants such as cell debris and apoptotic bodies [121]. Several commercial kits based on precipitation, including the Total Exosome Isolation Kit, ExoquickTM, Exoprep, miRCURRY, ExoGAG, Pure Exo, Exosome Precipitation Solution, and Total EV isolation reagent [122–124], are available for small extracellular vesicle (sEV) isolation. Precipitation-based EV isolation using commercially available reagents is a convenient and efficient method for processing clinical biological samples. However, one limitation is the potential for lower EV purity, as precipitation can also pellet proteins and lipoproteins along with EVs. Notably, precipitation reagents used in the isolation process may remain in EV preparations, impacting the viability and biological activity of recipient cells during downstream applications [125].

Affinity-based isolation of EVs relies on immunoaffinity capture assays that exploit specific surface proteins and receptors expressed on EVs. The mentioned techniques suggest yield, specificity, and integrity in the recovery of EVs from biological fluids [126, 127]. Immunoaffinity methods are easy to execute, fast, and compatible with routine laboratory equipment. However, the availability of antibodies and the presence of markers in the entire EV population can affect immunoaffinity capture assays [128]. To increase the selectivity, sensitivity, and yield of EV isolation, diverse immunoaffinity capture techniques utilizing microtiter plates, affinity columns, or magnetic beads have been established [121, 129]. Affinity-based EV isolation techniques using microfluidic chips provide advantages such as capturing and analyzing EVs from small clinical samples, making them highly suitable for liquid biopsy diagnosis [130]. However, it is important to consider the pros and cons of various EV isolation techniques.

Recent advancements in EV isolation and detection methods have led to the introduction of new approaches, particularly microfluidic platforms that utilize size-based separation, immunoaffinity-based separation, and dynamic separation techniques [131, 132]. These microfluidic systems offer advantages such as high purity, cost-effectiveness, and portability. Nevertheless, challenges such as complicated photolithography fabrication and the limitation of capturing EVs with only targeted proteins persist [105, 133]. Despite the development of novel tools, there is currently no standardized method for EV isolation or analytic technique. All of these techniques possess merits and demerits, underscoring the significance of choosing a suitable EV isolation approach contingent on specific research goals and criteria.

### Characterization of EVs

Characterization of EVs is a critical step in ensuring their identity, purity, and quality. To assess the physical and biochemical properties of EVs, a range of analytical techniques have been employed.

Nanoparticle tracking analysis (NTA) and dynamic light scattering (DLS) are commonly used methods for determining the size distribution and concentration of EVs, providing valuable insights into their physical attributes. Furthermore, protein marker analysis utilizing techniques such as Western blotting or flow cytometry allows researchers to evaluate specific EV-associated markers, such as TSG101, CD63, and ALIX, among others, to provide a better understanding of EV content and cargo. To visualize the morphology of EVs, electron microscopy techniques such as transmission electron microscopy (TEM), scanning electron microscopy (SEM), and atomic force microscopy (AFM) are used to obtain high-resolution images of EV structures.

Given that EVs are inherently heterogeneous, it is crucial to develop reliable methods for characterizing and validating their purity and cargo content. In addition to the aforementioned techniques, enzyme-linked immunosorbent assay (ELISA), fluorescence-activated cell sorting (FACS), resistive pulse sensing, and electrochemical biosensors have been developed to efficiently analyze and quantify EVs [120, 134, 135]. These advanced methods offer researchers a comprehensive toolkit for exploring and understanding the intricate properties of EVs, facilitating their potential applications in drug delivery and other therapeutic interventions.

### Current challenges of large-scale production of EVs

EVs have garnered significant attention in the field of RA research due to their potential therapeutic applications. However, the large-scale production of EVs for industry manufacturing in RA faces several challenges that need to be addressed [136]. One of the primary challenges is the standardization of EV isolation and purification methods to ensure consistent quality and yield. Variability in isolation techniques can impact the efficacy and safety of EV-based therapies, highlighting the need for standardized protocols [137]. Another challenge in promoting the large-scale production of EVs for rheumatoid arthritis is the scalability of production processes [138]. Current methods for isolating and purifying EVs are often labor-intensive and time-consuming, limiting their scalability for industrial manufacturing. Due to the financial constraints, technical complexities, and absence of appropriate biomarkers for specific exosomes, the isolation of significant amounts of pure and distinct exosomes from heterogeneous vesicle mixtures within a substantial solution volume poses a challenging task [139]. Developing efficient and cost-effective production methods that can be scaled up to meet the demand for EV-based therapies is crucial for advancing the field. To address isolation and purity concerns, manufacturing practices are transitioning away from time-consuming methods like UC, which may introduce contaminants. Instead, there is a shift towards scalable isolation techniques such as tangential flow filtration (TFF) or SEC [140].

Furthermore, the characterization and quality control of EVs pose challenges in ensuring the safety and efficacy of EV-based therapies for rheumatoid arthritis. Standardized methods for characterizing EV cargo, size distribution, and surface markers are essential for quality control and regulatory approval [141]. Addressing these challenges will require collaboration between researchers, industry partners, and regulatory agencies to establish guidelines for EV characterization and quality control. Further characterization of the composition of exosomes generated through manipulation of cellular origin or conditions is necessary [142]. These methods have the potential to impact the biological function of exosomes, potentially leading to fundamental alterations in cells that are not yet fully understood and may introduce new and undefined risks to research subjects. Enhancing the quantity and quality of exosomes requires the implementation of more effective isolation and purification methods. To facilitate the large-scale production of clinical-grade exosomes, researchers may need to integrate multiple methods to establish standardized and consistent quality procedures in the future [143].

Regulatory considerations also pose challenges for the large-scale production of EVs for rheumatoid arthritis. The regulatory landscape for EV-based therapies is still evolving, with varying requirements across different regions [144]. In the process and product development stages, careful planning and evaluation are essential for achieving realistic batch sizes for therapy in a clinical setting. When utilizing human material to produce EV-based therapeutics, a risk-based approach must be taken to assess the advantages of allogeneic or autologous use. In the context of larger-scale production, allogeneic strategies may be considered more favorable due to their scalability and accessibility [145]. Establishing master and/ or working cell banks to ensure a consistent supply of producer cells for EVs can be accomplished using media and supplements containing xenogeneic, human, or chemically defined materials [146, 147]. Safety concerns often lead to the preference for human-derived materials like pooled human platelet lysate, while scalability issues may favor chemically defined media [148]. Establishing clear guidelines for the production, characterization, and clinical use of EVs is essential for advancing the field and ensuring patient safety.

In conclusion, addressing the current challenges in promoting the large-scale production of EVs for industry manufacturing in RA is crucial for realizing the full potential of EV-based therapies. The current strategies for promoting the large-scale production of extracellular vesicles in industry manufacturing include advancements in bioprocessing technologies, such as bioreactor systems and cell culture techniques, to optimize the production efficiency of extracellular vesicles. A balance between purity, safety, and bioactivity is key to successful EV applications [149]. Additionally, standardizing isolation methods, improving scalability, enhancing characterization and quality control, and navigating regulatory considerations require attention and collaboration within the scientific and medical communities. By overcoming these challenges, we can accelerate the development and translation of EV-based therapies for the treatment of rheumatoid arthritis.

### EVs preservation and storage

Given the EVs critical role in signal transfer in a broad variety of physiological and pathological processes, many studies have shown EVs can promote tissue repair and regeneration in animal models including wound healing, diabetes, kidney injury, cardiac ischemia, and many others. Therefore, they have been considered to be used as drugs, drug carriers, and biomarkers [150].

Regarding the unique aspects of EVs, it’s crucial to identify preservation and cold chain strategies to translate preclinical findings into medical applications.

To preservation and store these vesicles, several conventional methods have been applied. The gold standard and widely accepted method for EV storage is keeping at -80 °C (cryopreservation), However, some issues such as expensive freezers as well as necessities related to maintaining the cold chain from the production to the patient make this method challenging. Furthermore, some investigations demonstrate that storage at -80°C cannot optimally preserve EVs and induces a loss of function in EVs [151].

Cryopreservation with cryoprotectants (CPAs) as another accepted method for long-term storage has been shown to maintain protein stability and prevent osmotic damage. To achieve ideal EV dehydration, it is essential to use cryoprotective agents (CPAs) to increase viscosity, influence ice nucleation kinetics, and allow controlled extracellular ice growth during controlled cooling. However, using extremely low CPA concentrations can result in chilling shock, which is the damage caused by the freezing process. Conversely, using excessively high CPA concentrations can be harmful. Therefore, finding the right balance is crucial for optimal cryopreservation other methods, such as freeze-drying (also known as lyophilization) and spray-drying, have been proposed as potential alternatives to the frozen storage of EVs [152].

Lyophilization involves freezing EVs, and the cooling rate affects ice crystal size. Sublimation then converts the frozen material into water vapor. However, freezing and dehydration stresses can damage EV biomolecules, requiring the use of CPAs for protection. Lyophilized EVs have extended shelf life, reduced storage needs, and lower costs. Stabilizers like glucose, lactose, sucrose, and trehalose are commonly used. Trehalose is suggested as the most effective disaccharide for preserving EVs during lyophilization. This technique is FDA-approved for proteins, liposomes, and nanoparticles, making it suitable for the pharmaceutical industry [153].

The spray drying process involves converting a solution containing EVs into a dry powder using heated gas. This continuous process can be automated and controlled for stability. The reduction in moisture content increases the stability of the biopharmaceuticals. Critical process parameters such as the feeding rate, atomization pressure, and outlet temperature must be carefully maintained. Further investigation is needed to broaden the application of this technique in manufacturing and storing EV-based therapeutics [154].

Both the storage of freshly isolated EVs and the recovery of EVs from previously stored biological samples seem to affect the physical and chemical properties of the particles. In 2013, the International Society for Extracellular Vesicles (ISEV) recommended preserving the samples at − 80 °C. They further specified to store EVs in phosphate-buffered saline (PBS) in siliconized vessels. However, in the 2018 update of ISEV guidelines, standard indications for EVs storage were not provided anymore [155].

Research has shown the potential of EVs in both research and clinical applications. However, there is no global consensus and standard operating protocol on the optimal preservation and storage conditions for these beneficial vesicles.

Several investigations have been carried out to ascertain the most optimal storage conditions for EVs. In the context of therapeutically intended EVs, research indicates that EVs derived from human embryonic kidney (HEK) 293T cells, endothelial colony-forming cells (ECFCs), and MSCs exhibit stability at temperatures as low as − 20 °C. These findings are consistent with the recommended standard storage temperature for EVs by ISEV. Conversely, an alternative study proposes that − 70 °C represents the most suitable long-term storage temperature for EVs isolated using the Exo-Quick kit.

Freeze–thaw stress is considered another challenge in the EVs storage. Repeated freeze–thaw cycles may affect the structural stability of EVs due to the exposure of vulnerable phosphatidylserine. This is important to consider as EV-based therapeutics are being developed, to ensure a clear product stability profile as required by regulatory bodies.

Different methods have been applied to reduce unwanted effects on EVs stability during preservation and storage. A bioengineering approach to overcome aggregation in EV Preparations could be one of these methods. The preparation of EVs can be considered as a colloid, and strategies to prevent EV aggregation involve modifying factors to increase interparticle repulsion and stabilize the colloidal solution. While PEG is commonly used for liposome stabilization, it is unsuitable for EVs, but coating the particles in polymer or using trehalose has been effective for EV preservation. Moreover, the addition of trehalose to EV solutions has been shown to enhance colloidal stability and improve particle yield. Biomaterial Scaffolds also can be used to enhance EV delivery and stability [156, 157].

The body's tissue matrix contains vesicles called matrix-bound vesicles (MBVs), which play a crucial role in enhancing their stability and availability. While there is still debate about whether MBVs possess all the characteristics of an EV, there is evidence that EVs can bind to ECM components. For example, a study showed that MSC-derived EVs bind to fibronectin and collagen type I in the ECM. Interaction with the matrix has been shown to enhance the stability of MBVs, and incorporating EVs with ECM or biomaterial components could be a powerful tool to provide controlled release within the body. Early studies have shown promising results in incorporating EVs into biomaterial constructs for delivery, demonstrating their potential for therapeutic applications [158].

## Different potential applications of EVs in the treatment of RA

### Biomarker potential of EVs in RA

EVs have demonstrated significant promise as potential biomarkers for RA. Although traditional clinical markers for RA have limited accuracy, the specific composition and content of EVs in the blood or synovial fluid (SF) can reflect disease conditions, making them promising candidates for diagnostic and monitoring purposes [159]. For example, RA patients with IgM-rheumatoid factor (IgM-RF) in their EVs exhibited increased disease activity, while the C-reactive protein (CRP) level and erythrocyte sedimentation rate (ESR) were elevated in the EVs of RA patients without IgM-RF [160]. This finding suggested a potential association between the presence of IgM-RF on EVs and increased disease activity in RA patients. Moreover, the expression levels of specific miRNAs, such as miR-155 and miR-146a, and the long noncoding RNA Hotair in blood cells and serum EVs from RA patients have been associated with disease progression and the migration of active macrophages in RA [161, 162]. Additionally, EVs derived from the synovial fluids of RA patients have been found to carry membrane-bound tumor necrosis factor (TNF) and citrullinated proteins, which contribute to disease progression [163, 164].

Various types of EVs, including CD146 + , CD41 + , CD66b + , CD14 + , CD3 + , CD4 + , CD8 + , CD161 + , CD39 + , CD73 + , CD105 + , annexin V + /CD45 + , and platelet-derived EVs, have been examined for their correlation with disease duration, age at diagnosis, serological markers, disease activity, and extra-articular symptoms. Associations have been observed between RF and various EV subtypes, while other studies have reported differences in EV profiles between patients with different serological RA phenotypes. Furthermore, lower levels of certain EVs have been associated with higher RF levels, and anti-citrullinated protein antibody (ACPA)-positive patients have shown higher levels of specific EV subtypes. EVs have also been linked to extra-articular symptoms and cardiovascular risk factors in RA patients [165].

In conclusion, EVs have emerged as important biomarkers in RA due to their ability to reflect disease conditions and carry specific cargo. The identification and analysis of EVs from RA patients have provided valuable insights into disease pathology and potential therapeutic targets. Utilizing EVs as biomarkers has the potential to improve the diagnosis, monitoring, and treatment of RA, ultimately enhancing patient outcomes and quality of life.

### Immunoregulatory effects of EVs in RA

EVs play important immunoregulatory roles in RA, particularly in modulating immune responses and reducing inflammation. Numerous studies have demonstrated the immunomodulatory effects of MSC-EVs on both innate and adaptive immune cells. For example, Mokarizadeh et al. showed that MSC-EVs contain programmed death-ligand 1, galectin-1, and TGF-β1; promote immune tolerance; and inhibit autoreactive lymphocyte proliferation, which promotes secretion of anti-inflammatory cytokines such as IL-10 and TGF-β. They also increase the expression of several regulatory molecules, specifically PD-L1 and TGF-β [166]. In another study, Cho et al. investigated the immunosuppressive functions of microparticles (MPs) and exosomes derived from MSCs. Both MPs and exosomes effectively suppressed anti-inflammatory cells, such as CD4 + IL-10 + Tr1 cells and CD4 + Tregs, in a dose-dependent manner and increased the number of pro-inflammatory cells, such as CD8 + IFN-γ + cells. These EVs were also found to influence macrophage maturation, resulting in decreased levels of TNFα and increased levels of IL-10. Furthermore, in the CIA model, exosomes significantly reduce arthritis symptoms by inhibiting plasmablast differentiation and inducing IL-10 production in Breg cells [167]. Additionally, EVs secreted by human UC-MSCs have been shown to suppress T-cell proliferation, stimulate the apoptosis of T cells, regulate the balance of Tregs and Th17 cells, and inhibit synovial hyperplasia both in vitro and in vivo [61].

Additionally, studies have revealed that EVs obtained from immature dendritic cells (DCs), which are generated through genetic manipulation or cytokine inhibition, possess immunosuppressive and tolerogenic characteristics. These findings hold promise for regulating both adaptive and innate immune responses. Various types of EVs derived from DCs have been investigated for their therapeutic potential in suppressing RA-related immune responses. For instance, EVs derived from IL-4-expressing DCs have shown similar effectiveness in reducing the severity and occurrence of CIA when administered systemically or locally. The suppressive effects of these EVs are MHC-restricted, and they can directly or indirectly modify the function of endogenous antigen-presenting cells (APCs) and T cells. These EVs induce a regulatory subset and/or deplete antigen-reactive Th1 cells [168, 169]. Another approach involves utilizing EVs derived from DCs treated with IL-10. These IL-10-treated DC-derived EVs have been found to suppress inflammatory and autoimmune responses by inhibiting proinflammatory cytokines and reducing the levels of the heat shock protein Hsp70. For instance, Kim et al. demonstrated that the systemic administration of IL-10-treated DC-derived EVs inhibits disease progression and decreases the severity of arthritis in CIA animal models [170]. Furthermore, in a murine model of DTH, EVs derived from DCs expressing Fas ligand (FasL) demonstrated anti-inflammatory properties. The therapeutic effects of these EVs were specific to the target antigen and dependent on MHC class II molecules. Systemic administration of these EVs has shown significant effectiveness in treating the established CIA mouse model [171, 172]. In addition, indoleamine-pyrrole 2,3-dioxygenase (IDO)-expressing DCs can secrete EVs with immunosuppressive properties. These EVs have increased stability and bioactivity for efficient delivery. Bianco et al. reduced inflammation through direct interactions with T cells via the costimulatory molecules B7-1 and B7-2, independent of tryptophan availability, in the CIA and DTH disease model [90].

Moreover, the presence of annexin A1, an inhibitory mediator of arthritis, has been detected in EVs derived from neutrophils in the synovial fluid of RA patients. Moreover, in animal models, annexin A1 plays a role in the anti-inflammatory and chondroprotective effects of neutrophil-derived EVs through interactions with its receptor FPR2. These interactions induce anabolic responses, such as TGF-β1 production and extracellular matrix deposition, while protecting chondrocytes from apoptosis. Annexin A1 is also a component of EVs generated from adipose tissue-derived MSCs, potentially contributing to their anti-inflammatory properties [77, 173].

It has also been shown that EVs carry a diverse range of functional molecules, such as proteins, lipids, miRNAs, and long noncoding RNAs (lncRNAs), and that their functions are influenced by these cargo molecules [174]. Research has notably illustrated the crucial role of specific miRNAs transported by EVs in diminishing inflammation and regulating immunity in RA patients [175]. Chen et al. demonstrated that MSC-derived exosomes carrying miR-150-5p (Exo-150) have therapeutic potential for mitigating joint destruction in RA. Notably, Exo-150 significantly inhibited cell migration and infiltration of RA patient-derived FLSs; inhibited tube formation in human umbilical vein endothelial cells (HUVECs); and attenuated synoviocyte hyperplasia and angiogenesis in a CIA mouse model [55]. Specific EV miRNAs, such as miR-451a and miR-223-3p, which are enriched in the joints of RA patients with low-grade and high-grade inflammation, respectively, have been shown to significantly regulate joint inflammation. MiR-451a suppresses inflammation by inhibiting Akt/mTOR pathway activation, cytokine expression, and T-cell activation In contrast, miR-223-3p plays dual roles in promoting osteoclast differentiation while also protecting against inflammatory arthritis in mouse models [176]. Overall, EVs have shown significant immunomodulatory effects in RA by modulating the function of immune cells, suppressing inflammatory responses, promoting the production of regulatory cells, and protecting against joint destruction.

### EV-based targeted drug delivery for RA

EVs have emerged as a promising and reliable tool for drug delivery in RA treatment. Encapsulating drugs within EVs shields them from enzymatic degradation and enables specific delivery to the intended target [177]. EV-based drug delivery has already demonstrated positive outcomes in treating various diseases, including brain inflammation and cancer [178, 179].

To optimize the effectiveness of EVs as drug carriers in RA treatment, researchers have devised various strategies to enhance their loading capacity, stability, targeting efficiency, and overall therapeutic efficiency [180, 181]. One such strategy involves loading EVs with drugs or biomolecules using physical or chemical methods, ensuring efficient encapsulation of therapeutic agents. For instance, a study demonstrated the potential of utilizing dendritic cell-derived EVs to encapsulate triptolide (TP), mitigating TP-induced toxicity while inducing immunosuppression in murine models of ulcerative colitis and rheumatoid arthritis (RA) [182]. Furthermore, MSC-derived exosomes loaded with miR-320a effectively regulate fibroblast-like synoviocytes (FLSs) in rheumatoid arthritis (RA), suppress CXCL9 expression and inhibit RA-FLS activation, migration, and invasion in vitro while attenuating arthritis and bone damage in a CIA mouse model [54].

Another approach involves modifying the surface of EVs using ligands or antibodies capable of binding to specific receptors on target cells for targeted drug delivery. For instance, macrophage-derived EVs encapsulating Dex nanoparticles (EVs/Dexs) were functionalized with a compound consisting of folic acid (FA), polyethylene glycol (PEG), and cholesterol (Chol), resulting in FPC-EVs/Dexs. These engineered EVs exhibited enhanced anti-inflammatory and immunomodulatory effects both in vitro and in vivo [101]. Additionally, metabolic glycan engineering (MGE) coupled with click chemistry was utilized to produce EVs from ADSCs, resulting in the formation of engineered EVs that efficiently accumulated in the inflamed joints of mice with collagen-induced arthritis, inducing anti-inflammatory events through macrophage phenotype regulation. Notably, these engineered EVs demonstrated therapeutic efficacy comparable to that of bare exosomes but required significantly lower dosages [183]. Moreover, primary M2 macrophage-derived EVs modified with cell-penetrating peptide (R9) exhibited enhanced uptake by target cells and inflammation targeting. Loading these EVs with curcumin has been shown to have prominent anti-inflammatory effects on SCI and RA mouse models, promoting macrophage repolarization toward the anti-inflammatory M2 phenotype in vitro [100].

In parallel, researchers have explored the genetic manipulation of donor cells to optimize the composition or function of released EVs for improved therapeutic outcomes. For instance, genetically engineered small EVs carrying IL4 exhibited stronger anti-inflammatory effects on M1-polarized macrophages, promoting M2 polarization more effectively than soluble IL4 proteins in a collagen-induced arthritis model [184]. Another study involved EVs produced from M2 macrophages transfected with IL-10 plasmid DNA (IL10pDNA) and encapsulated in betamethasone sodium phosphate (BSP), a chemical drug. In mice with collagen-induced arthritis, BSP-IL10pDNA-EVs showed potent anti-inflammatory activity at the joint site, leading to increased body weight and reduced paw swelling [184]. Additionally, miR-146a/miR-155-modified MSC-derived EVs exhibited potent effects on regulatory T cells and anti-inflammatory cytokines in a murine model of CIA, suggesting their potential as a potentially effective therapeutic approach for RA [185].

**Fig. 5:**
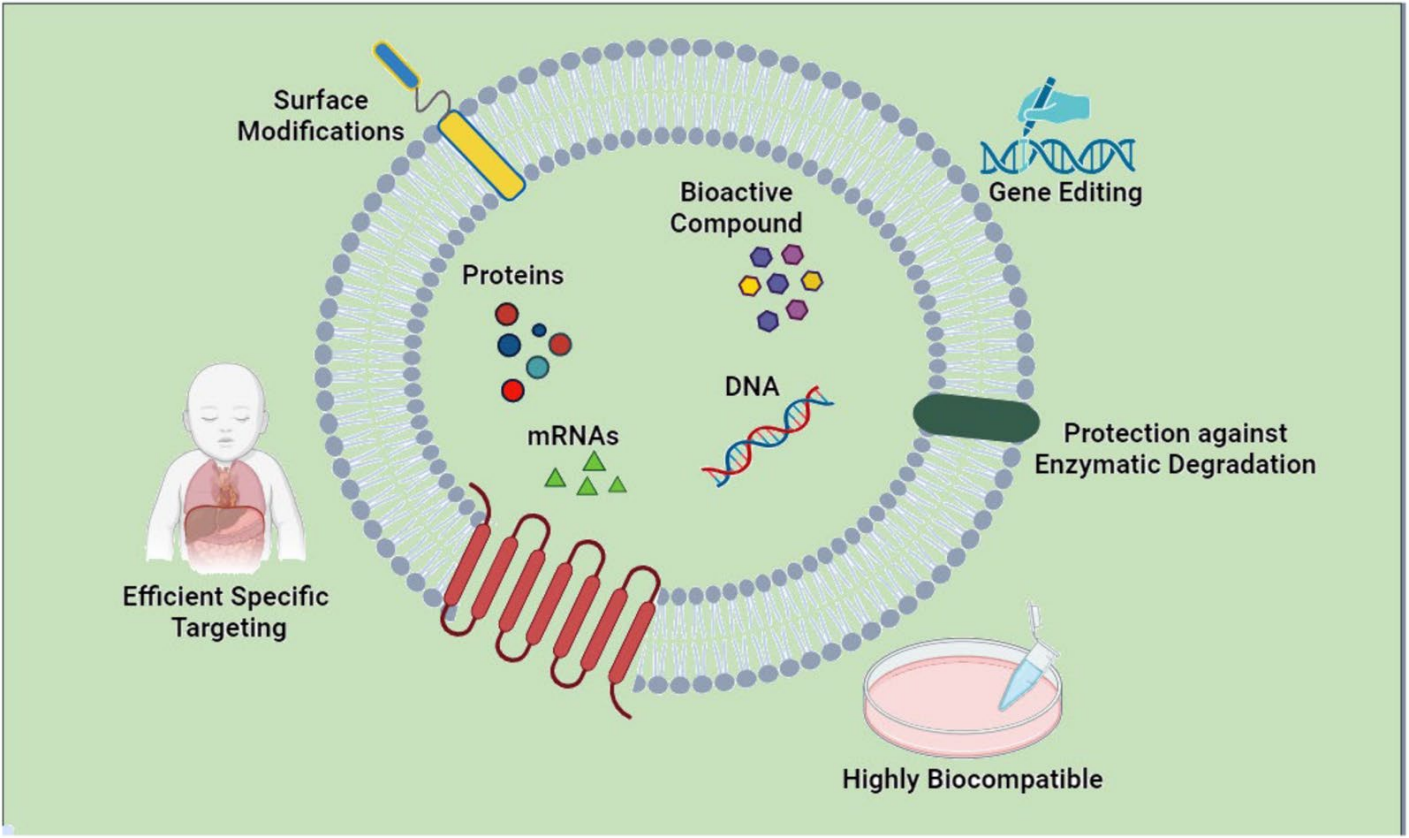
The advantages of targeted drug delivery in RA. The new drug delivery systems based on EVs are currently working to enhance drug delivery, promote better targeting, and reduce the toxicity of conventional antifungal drugs. (The figure Created by biorender.com)

In conclusion, EVs hold immense potential as drug carriers for RA treatment, and their optimization through innovative strategies such as surface modification and genetic engineering shows promise in targeted drug delivery (Fig. 5). These advancements pave the way for future developments in the field of EV-based therapies for RA. The list of total studies related to EV-based targeted drug delivery for RA and EV isolation methods is summarized in Table 2.

**Table 2:** EVs-based targeted drug delivery for RA

EV Sources	EV Type	Delivery Molecule	EV’s Modification	Effect	References
Dendritic cells (DCs)	Exosome	Triptolide	None	Reducing toxicity and promoting immunosuppression	[113]
hBM-MSCs	Exosome	microRNA-320a	None	Attenuating arthritis and bone damage	[17]
Macrophages	Exosome	Dexamethasone sodium phosphate	The surface was modified with FA- PEG- Chol	Enhancing drug delivery Improving anti-inflammatory effects Reducing side effects	[52]
ADSCs	Exosome	None	Surface modified by metabolic glycoengineering (MGE) in combination with bioorthogonal copper-free click chemistry	Inducing a polarization effect toward the anti-inflammatory macrophage phenotype (M2) in the inflamed joints Delivering therapeutic cargos to inflamed joints in a mouse model of RA	[114]
Macrophages	Exosome	Curcumin	Surface Modification with R9 peptide	Promoting the repolarization of macrophages in vitro Enhanced targeting and anti-inflammatory effect In vivo	[51]
Human embryonic kidney cells (HEK293)	Small extracellular vesicles (EVs)	IL-4	Transfected HEK293 cells with IL-4 plasmid DNA encoding (pCMV-IL4-LA)	Anti-inflammatory effects on M1-polarized macrophages through enhanced the M2 polarization Amelioration of chronic inflammation In vivo	[115]
Macrophages	Exosome	Betamethasone sodium phosphate (BSP)	Transfected M2 macrophages with IL-10 pDNA	Reducing inflammation and promotion of M1-to-M2 macrophage polarization in vitro Accumulation at inflamed joint sites and high anti-inflammatory activity In vivo	[116]
mBM-MSCs	Exosome	miR-146a/miR-155	Transduced MSCs with miR-146a/miR-155	Alteration of Treg cell levels and modulation of gene expression associated with anti-inflammatory and pro-inflammatory responses	[117]

## Conclusion, challenges, and future perspectives

This review provides a comprehensive overview of the emerging field of extracellular vesicle-based targeted therapies for RA, which requires novel therapeutic strategies to overcome the limitations of existing treatments. As small membrane-bound particles are produced by various cell types, our literature has described the ability of EVs to modulate immune responses in the inflamed environment of damaged joint tissues. Furthermore, we identified MSCs, neutrophils, granulocytic myeloid-derived suppressor cells, dendritic cells, and macrophages as the main sources of EVs. Each source confers unique characteristics and therapeutic potential, adding to the complexity of the evolving field of EV-based therapies. Despite the potential benefits of EV-based therapies, it is essential to acknowledge the significant challenges associated with this domain. The pathophysiology of RA is complicated, and current treatment strategies rely primarily on anti-inflammatory drugs, which often cause adverse side effects and provide only temporary relief, thereby hindering disease progression. Despite the development of promising antibodies and compounds, a definitive cure for RA has not been identified, necessitating a shift toward combination therapies, especially in advanced RA stages. In particular, EV-based targeted therapies show promise as a new approach for the treatment of RA, although several challenges need to be further addressed. These include standardization of isolation and characterization methods, determination of optimal dosing and treatment regimens, and clarification of long-term safety and efficacy. Future research should focus on refining EV isolation techniques, unraveling the mechanisms underlying EV-mediated therapeutic effects, and conducting rigorous clinical trials to establish the efficacy of EV-based therapies in RA patients. Currently, advances in understanding the functions of EVs, especially their roles as potent anti-inflammatory agents and as mechanisms for targeted drug delivery, including antibodies, peptides, and miRNAs, have contributed to preclinical and clinical studies of RA treatment. This paper provides an overview of how these factors influence therapeutic approaches for RA and influence the choice of treatment methods and clinical outcome measures.

## Supplementary Information

Additional file 1.

## Data Availability

Not applicable.
