# Correction: Advancements in extracellular vesicle targeted therapies for rheumatoid arthritis: insights into cellular origins, current perspectives, and emerging challenges

**DOI:** 10.1186/s13287-024-04047-x

**Published:** 2024-11-07

**Authors:** Maryam Talebi Jouybari, Fatemeh Mojtahedi, Mahnaz Babaahmadi, Maryam Faeed, Mohammadreza Baghaban Eslaminejad, Leila Taghiyar

**Affiliations:** 1 Department of Stem Cells and Developmental Biology, Cell Science Research Center, Royan Institute for Stem Cell Biology and Technology, ACECR, Tehran, Iran; 2 Department of Developmental Biology, University of Science and Culture, Tehran, Iran; 3 Department of Immunology, Shahid Sadoughi University of Medical Science, Yazd, Iran; 4 School of Biology, College of Science, University of Tehran, Tehran, Iran; 5 Advanced Therapy Medicinal Product Technology Development Center (ATMP-TDC), Royan Institute for Stem Cell Biology and Technology, ACECR, Tehran, Iran

**Correction to: Stem Cell Research & Therapy (2024) 15:276**

10.1186/s13287-024-03887-x

The authors wish to correct the information for affiliation #1 to the following:

Department of Stem Cells and Developmental Biology, Cell Science Research Center, Royan Institute for Stem Cell Biology and Technology, ACECR, Tehran, Iran.

