# Supplementary material for: Advancements in extracellular vesicle targeted therapies for rheumatoid arthritis: insights into cellular origins, current perspectives, and emerging challenges
Source: Stem Cell Res Ther. 2024 Sep 4;15:276. doi: 10.1186/s13287-024-03887-x (PMC11373471; doi:10.1186/s13287-024-03887-x)
Supplement: Supplementary file 1 — Additional file 1. [file 13287_2024_3887_MOESM1_ESM.docx]

| Supplementary Table 1. Clinical trials with MSC/Cell therapy up to 2022 | | | | | | |
| --- | --- | --- | --- | --- | --- | --- |
| RA Patients | **Cell type** | **Doses** | **MHC Context; Route of Administration** | **Phase of study** | **Outcome** | **Clinical Trial**  **Identifier** |
| Refractory RA | AD-MSC | 1, 2 or 4 ×10^6^  3 doses, weekly | Allogeneic; IV | Phase Ib/IIa | safe and well-tolerated  DAS28-ESR↓, CRP↓,  ACR20 response after 1 month (20–45%) and 3 months (15–25%) | NCT01663116 |
| RA | BM-MSC | 4×10^7^ per joint | Autologous; IA | phase 1/2 | safe and well-tolerated  DAS28↓ (NS), VAS↓, WOMAC↓, ESR↓ (NS), CRP↓ (NS), Pain FWD↑, WD↑, Time to jelling↑, Standing time↑ | NCT01873625 |
| Refractory RA | BM-MSC | 1 to 2×10^6^ cells/Kg  1 dose | Autologous; IV | Phase 1 | safe and well-tolerated  DAS28-ESR↓, VAS↓, ESR↓, CRP↓(NS), RF↓, anti-CCP↓ (NS) | NCT03333681 |
| Refractory RA | UC-MSC | 2.5, 5 or 10 × 10^7^/patient;  1 dose | Allogeneic; IV | Phase 1 | safe and well-tolerated  DAS28↓, VAS↓, HAQ↓, CRP↓, IL-1β↓, IL-6↓, IL-8↓, TNF-α↓ | NCT02221258 |
| Refractory RA | UC-MSC | 4 × 10^7^/patient;  1 dose | Allogeneic; IV | phase 1/2 | safe and well-tolerated  DAS28↓, HAQ↓, CRP↓, ESR↓, RF↓, anti-CCP↑, TNF-α↓, IL-6↓ | NCT01547091 |
| Refractory RA | UC-MSC | unreported | Allogeneic; IV | phase 1/2 | unreported | NCT01985464 |
| RA | BM-MSC | unreported | Autologous, IV, and IA | Phase 1 | unreported | NCT03067870 |
| RA | UC-MSC | 1×10^6^  1 dose | Allogeneic; IV | NA | unreported | NCT03798028 |
| RA During onset | MPCs | 2, 4, or 6 × 10^6^;  1 dose | Allogeneic; IV | Phase 1 | unreported | NCT03186417 |
| RA | UC-MSC | 2×10^7^ | Allogeneic; IV |  | unreported | NCT02643823 |
| Refractory | UC-MSC | 10 × 10^7^/patient;  3 doses | Allogeneic; IV | phase 1/2 | unreported | NCT03618784 |
| Active RA | AD-MSC | Unknown | Autologous;IV | phase 1/2 | safe and efficacious for improvement in joint function | NCT03691909 |
| Refractory RA | UC-MSC | 0.75 or 1.5 ×10^6 ;^ 1 dose | Allogeneic; IV | Phase 1 | unreported | NCT03828344 |
| Refractory RA | AT-MSC | 2.0 or 2.86 ×10^6^;  1 dose or 3 doses, every 3 days | Autologous;IV | phase 1/2a | unreported | NCT04170426 |
| RA | AT-MSC | Unknown | Autologous; IV | Pilot | VAS↓, KWOMAC↓, CRP↓, RF↓, anti-CCP↓, standing time↑, WD↑, off steroids | NCT01413061 |
| RA | tDC | 1×10^6^, 3×10^6^, 5×10^6^, 8×10^6^ and 10×10^6^ cells in 2.0 mL sodium chloride 0.9% solution) | Autologous; IA | Phase 1 | unreported | NCT03337165 |
| RA | UC-MSC | 100×10^6^ | Allogeneic; IV | Phase 1 | unreported | NCT05003934 |
| AT, adipose tissue; BM, bone marrow; UC, umbilical cord; MPCs, mesenchymal progenitor cells; IV, Intravenous; N/A; Not applicable; IA, intra-articular; tD, Tolerogenic dendritic cell; Western Ontario and McMaster Universities Arthritis Index (WOMAC); Korean Western Ontario and McMaster Universities Arthritis Index (KWOMAC); visual analog scale (VAS); the American College of Rheumatology criteria (ACR); Health Assessment Questionnaire (HAQ); disease activity score 28 (DAS28); pain-free walking distance (Pain FWD); walking distance (WD); erythrocyte sedimentation rate (ESR); C-reactive protein (CRP); rheumatoid factor (RF); anti-cyclic citrullinated antibody (anti-CCP); non-significant (NS); increasing level (↑); decreasing level (↓). | | | | | | |

| Supplementary Table 2. Clinical trials in gene therapy for Rheumatoid Arthritis | | | | | |
| --- | --- | --- | --- | --- | --- |
| Transgene | **Delivery method** | **Study phase** | **Sponsor/Principal Investigator** | **Subjects number** | **Reference** |
| IL-1Ra | Retrovirus  Ex-vivo | I | University of Pittsburgh (Evans and Robbins) | 9 | (27) |
| IL-1Ra | Retrovirus  Ex-vivo | I | University of Dusseldorf  (Wehling) | 2 | (28) |
| Etanercept | AAV  In-vivo | I/II | (Mease) | 127 | (29) |
| ART-I02 | recombinant adeno-associated virus (AAV) | I | Arthrogen  Netherland | 12 | (30) |

| Supplementary Table 3. Clinical Study in Tissue Engineering for RA | | | | | |
| --- | --- | --- | --- | --- | --- |
| Study Title | **Study location** | **Study Phase** | **No. of patients** | **Outcomes** | **Refs.** |
| [Safety of Cultured Allogeneic Adult Umbilical Cord Derived Mesenchymal Stem Cell Intravenous Infusion for RA](https://clinicaltrials.gov/ct2/show/NCT05003934?term=regenerative+medicine&cond=Rheumatoid+Arthritis&draw=2&rank=1) | Mexico and Argentina | I | 20 | N/A | (30) |
| [Transplantation of Bone Marrow-Derived Mesenchymal Stem Cells in Affected Knee Osteoarthritis by Rheumatoid Arthritis](https://clinicaltrials.gov/ct2/show/NCT01873625?term=transplantation&cond=Rheumatoid+Arthritis&draw=2&rank=1) | Iran | II | 60 | N/A | (31) |
| [Rheumatoid Arthritis: Tolerance Induction by Mixed Chimerism](https://clinicaltrials.gov/ct2/show/NCT00282412?term=transplantation&cond=Rheumatoid+Arthritis&draw=2&rank=5) | United States | I | N/A | No participant enrolled for three years due to termination of this study | (32) |
| [Stem Cell Support in Patients with Rheumatoid Arthritis](https://clinicaltrials.gov/ct2/show/NCT00278551?term=transplantation&cond=Rheumatoid+Arthritis&draw=2&rank=6) | United States | I | 10 | N/A | (33) |
